# The Protective Effects of 18*β*-Glycyrrhetinic Acid on* Helicobacter pylori*-Infected Gastric Mucosa in Mongolian Gerbils

**DOI:** 10.1155/2016/4943793

**Published:** 2016-02-24

**Authors:** Donghui Cao, Jing Jiang, Lili You, Zhifang Jia, Tetsuya Tsukamoto, Hongke Cai, Shidong Wang, Zhen Hou, Yue-er Suo, Xueyuan Cao

**Affiliations:** ^1^Division of Clinical Epidemiology, First Hospital of Jilin University, Changchun 130021, China; ^2^Division of Pathology I, School of Medicine, Fujita Health University, Toyoake 470-1192, Japan; ^3^Department of Gastric and Colorectal Surgery, First Hospital of Jilin University, Changchun 130021, China

## Abstract

18*β*-Glycyrrhetinic acid (GRA), a major component of* Glycyrrhiza glabra*, is widely used therapeutically in clinic. In this study, the effect of GRA on* Helicobacter pylori*- (*H. pylori*-) infected gastritis was investigated in Mongolian gerbils* in vivo*. The gerbils were randomly divided into groups: uninfected;* H. pylori*-infected;* H. pylori *+ antibiotics (clarithromycin, amoxicillin, and esomeprazole); and* H. pylori* + GRA. The gastric intraluminal pH value, histopathological changes, and the expression levels of inflammation-related cytokines (IL-1*β*, TNF-*α*, COX-2, and iNOS) were investigated. The results showed that, in the* H. pylori *+ GRA group, the intraluminal gastric pH value was lower (2.14 ± 0.08 versus 3.17 ± 0.23, *P* < 0.05), erosion and hyperplasia were alleviated, the infiltration of neutrophils and mononuclear cells was attenuated (*P* < 0.05), and the expression levels of TNF-*α*, IL-1*β*, COX-2, and iNOS were decreased (*P* < 0.05) compared with the* H. pylori*-infected group. There was no significant difference in results between the* H. pylori *+ GRA group and the* H. pylori *+ antibiotics group. This study indicated that GRA significantly attenuated* H. pylori*-infected gastritis in gerbils and has the potential to be developed as a new therapeutic drug.

## 1. Introduction

Gastric cancer is one of the most common malignancies, ranking fifth highest in incidence and third highest in terms of cancer-related death worldwide [1]. Many epidemiological studies have shown that* Helicobacter pylori* (*H*.* pylori*) is closely involved with gastric cancer [2, 3], and it has been designated a class I gastric carcinogen by the World Health Organization (IARC, 1994) [4].* H*.* pylori* is the dominant species of the human gastric microbiome and specifically colonizes the gastric epithelium [5]. When infected by* H*.* pylori*, the gastric mucosa can develop various gastric diseases, from superficial gastritis producing mild discomfort to severe tissue disease, encompassing peptic ulcers, gastric cancer, or mucosa-associated lymphoid tissue lymphoma. Epidemiological data have shown that eradication of* H. pylori *could suppress gastric carcinogenesis in infected individuals [6]. Therefore, eradication of* H. pylori* appears to be the most direct approach to reducing gastric cancer. First-line treatment for* H. pylori *is triple therapy, the administration of a proton pump inhibitor, clarithromycin, and amoxicillin for 7–14 days [7]. Previous reports have shown that* H. pylori *has evolved antibiotic resistance to these agents [8]. New strategies to eradicate* H. pylori *infection, such as vaccine, probiotics, and nutraceuticals, are in development [9].

A large number of scientific publications worldwide have described the antibiotic activity of herbal products against* H. pylori. *Several traditional Chinese medicines, including canolol, radix curcumae,* Morinda citrifolia*, curcumin,* Polygonum capitatum*, and cinnamaldehyde, have been found to reduce the inflammation induced by* H. pylori* infection [10–16].

18*β*-Glycyrrhetinic acid (GRA), the main component purified from liquorice root (*Glycyrrhiza glabra*), is known for its antileukemic, anti-UV-B irradiation, anti-invasive, antiangiogenic, and antioxidation properties [17–22]. To date, there are no* in vivo *reports of the effects of GRA on inflammation caused by* H. pylori *infection, and the mechanisms of anti-inflammation needed to be elucidated.

The Mongolian gerbil is considered to be the stable and standard animal model to evaluate the effects of* H. pylori *infection [23, 24]. Mongolian gerbils are particularly suitable because they rarely develop gastritis unless infected with* H. pylori*; the colony-forming units of* H. pylori *needed to colonize Mongolian gerbils are considerably fewer than those needed in mice, and the histological changes, including gastric atrophy, ulcers, intestinal metaplasia, and adenocarcinomas closely resemble those seen in infected humans. This model has been widely used in research on gastritis [23], peptic ulceration [25], and gastric carcinogenesis [24, 26].

The main objective of this study was to determine the* in vivo* effects of GRA on the inflammatory responses caused by* H. pylori *infection and to discuss the mechanisms involved.

## 2. Materials and Methods

### 2.1. Animals

Six-week-old, male specific-pathogen-free Mongolian gerbils (*Meriones unguiculatus*; MGS/Sea), purchased from Zhejiang Medical Research Institute (Hangzhou, China), were maintained in an air-conditioned room with free access to a commercial rodent diet and water* ad libitum*.

### 2.2. Experimental Protocol

**Figure 1:**
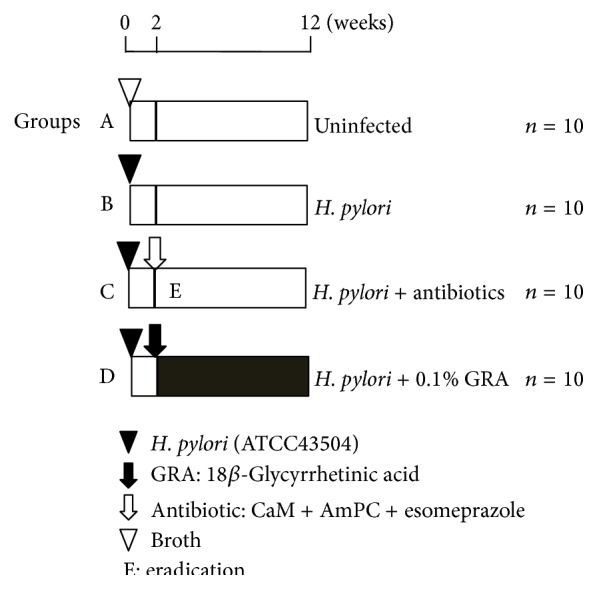
Experimental design.

After a week of acclimatization, the 40 7-week-old gerbils were divided into groups; 10 animals were used as control and were not infected with* H. pylori* (Group A); the remaining 30 gerbils were inoculated with* H. pylori*. Ten of these animals were given no treatment (Group B), 10 animals were treated with clarithromycin, amoxicillin, and esomeprazole (Group C), and 10 gerbils were treated with 0.1% GRA (Group D) (Figure 1). The gerbils were weighed every week. For treatments, all animals were subjected to deep anesthesia using diethyl ether and all efforts were made to minimize suffering. All experiments and procedures carried out on the animals were approved by the Animal Care Committee of the First Hospital of Jilin University.

### 2.3. *Helicobacter pylori* Culture and Infection

*Helicobacter pylori *ATCC 43504 (American Type Culture Collection, Manassas, VA, USA), a type I strain, were cultured in Brucella broth supplemented with 7% fetal calf serum at 37°C under microaerophilic conditions for 48 h. To establish* H. pylori *infection, Mongolian gerbils in Groups B, C, and D were inoculated three times with 0.8 mL of sterile broth culture containing 1 × 10^8^ colony-forming units of* H. pylori *by gastric intubation at 48 h intervals. The animals were fasted for 24 h before the first inoculation.

### 2.4. GRA Administration and Antibiotics Treatment

GRA purchased from Sigma-Aldrich (St. Louis, MO) was dissolved in distilled water at 0.1% concentration using ultrasonication. This solution was freshly prepared three times per week and administered as drinking water, starting 2 weeks after* H. pylori* infection and continuing for 10 weeks.

For eradication of* H. pylori*, therapy was similar to our previous study [27]: clarithromycin, amoxicillin, and esomeprazole were suspended in 0.5% w/w carboxymethyl cellulose sodium salt solution and administered intragastrically twice a day for 2 days at doses of 30, 3, and 10 mg/kg body weight, respectively.

### 2.5. Tissue Collection and Intraluminal pH Test

All gerbils were sacrificed 12 weeks after* H. pylori *infection and laparotomized, and the stomachs were resected and cut along the greater curvature. The pH value of the succus gastricus was tested using pH test strips. Bacterial colonization of* H. pylori* in Groups B, C, and D was assessed by homogenizing ~30 mm^2^ of stomach mucosa with 1.0 mL of phosphate-buffered saline. This solution was cultured for* H. pylori*: 100 *μ*L aliquots were inoculated onto* H. pylori *agar plates (Nissui Pharmaceutical, Tokyo, Japan), which were then incubated at 37°C under microaerophilic conditions. The number of* H. pylori *colonies was counted after 5–7 days [28].

The remainder of the sample was divided into two parts: one part was fixed in 10% neutral buffered formalin and paraffin embedded for hematoxylin-eosin (H&E) staining and histopathological analysis; the other part was immediately frozen in liquid nitrogen and stored at −80°C for RNA extraction.

### 2.6. Histopathological Analyses

The paraffin-embedded tissue was serially sectioned into 4 *μ*m thick sections, which were stained with H&E for histological analysis to detect any inflammatory and/or epithelial changes. Active chronic gastritis was characterized by infiltration of neutrophils and lymphocytes. The degree of inflammatory change, hyperplasia, and peptic ulceration was graded on a 4-point scale (0–3: 0: normal; 1: mild; 2: moderate; 3: marked) according to the criteria modified from the updated Sydney System [29].

### 2.7. RNA Extraction and mRNA Quantification

Total RNA was extracted using TRIzol reagent (Invitrogen Life Technologies, Carlsbad, CA, USA) according to the manufacturer's instructions, and RNA was stored at −80°C. cDNA was synthesized from 1 *μ*g total RNA using a High-Capacity cDNA Reverse Transcription Kit (Invitrogen Life Technologies). Expression levels of inflammation-related genes, TNF-*α*, IL-1*β*, COX-2, and iNOS, were analyzed using the reverse transcription products on a 7500 Fast Real-Time PCR system (Applied Biosystems, Carlsbad, CA, USA), with an initial denaturation at 95°C followed by 40 cycles of 95°C for 15 s and 60°C for 1 min. GAPDH was used as an internal control. A tube with no reverse transcriptase was included to control for any DNA contamination. The Applied Biosystems 7500 Fast software (Applied Biosystems) was used to analyze the CT values, and the 2^−ΔΔCt^ method for relative quantitation was used to determine the changes of mRNA expression. The primers used are listed in Table 1.

### 2.8. Statistical Analysis

All analyses were performed using SPSS^®^ version 10.0 (SPSS Inc., Chicago, IL, USA) or GraphPad Prism, version 5.0 (La Jolla, CA, USA). Data were evaluated using one-way analysis of variance. A value of *P* < 0.05 was considered significant and *P* < 0.01 was very significant.

## 3. Results 

All 40 gerbils survived to the experiment endpoint. The colonization rate of* H. pylori *was 100% in Groups B, C, and D. And, at the end of the study, the colonization rate was 0% in the antibiotic group (Group C). However, the number of* H. pylori *colonies was not affected in GRA treatment group (Groups D). There was no significant difference in body weights of the animals between the different groups (data not shown).

### 3.1. GRA Decreased Gastric pH Value Caused by* H. pylori* Infection in Mongolia Gerbils

**Figure 2:**
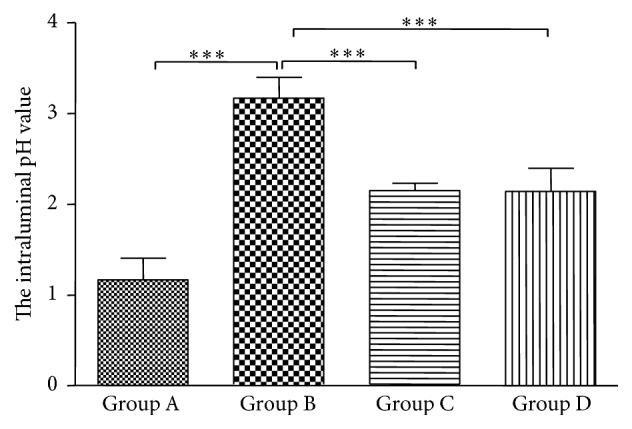
Gastric intraluminal pH values of Mongolian gerbils 12 weeks after infection with* Helicobacter pylori *and either antibiotics therapy treatment or 18*β*-Glycyrrhetinic acid (GRA); ^*∗∗∗*^ *P* < 0.001.

At the study end, the mean pH of the succus gastricus in Group A animals (uninfected controls) was 1.2 ± 0.2. After 12 weeks of* H. pylori *infection, mean succus gastricus pH was 3.2 ± 0.2 (Group B; *P* < 0.001, compared with Group A). Antibiotics treatment (Group C) produced a succus gastricus pH of 2.6 ± 0.1 (*P* < 0.001 compared with Group B) and treatment with GRA a value of 2.1 ± 0.3 (*P* < 0.001 versus Group B). This showed that GRA had a similar effect on the antibiotics treatment regimen in reducing the higher pH caused by* H. pylori *infection (Figure 2).

### 3.2. GRA Alleviated* Helicobacter pylori* Infection-Induced Gastritis

**Figure 3:**
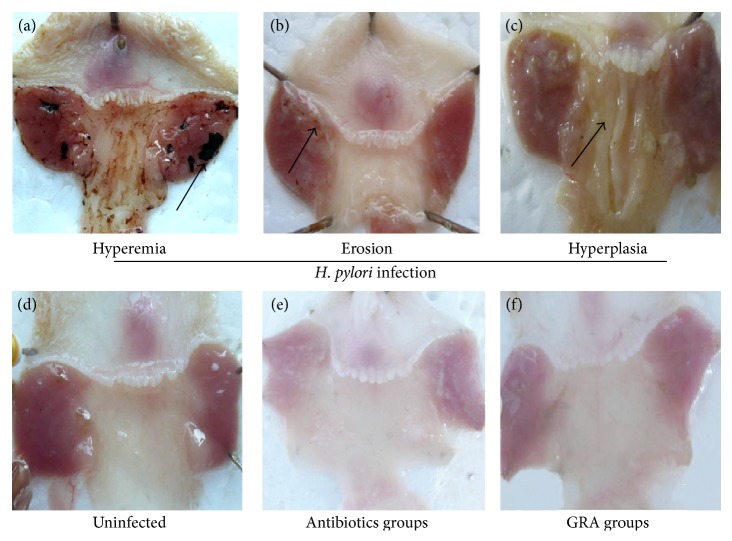
The macroscopic gastric mucosa findings in gerbils infected by* Helicobacter pylori* (upper panel: (a) hyperemia; (b) erosion; (c) hyperplasia), after antibiotics treatment (e) or 18*β*-Glycyrrhetinic acid (GRA) (f). Uninfected gerbils were controls (d).

Uninfected Mongolian gerbils had a smooth gastric mucosal surface (Figure 3(d)), but, after 12 weeks of* H. pylori *incubation, that smoothness was replaced by gastric mucosal hyperemia, edema, and erosion (Figures 3(a)–3(c)), and the hyperplasia and peptic ulcer score (Sydney System) were significantly increased (*P* < 0.001 for Group B compared with Group A). The Sydney System score was less with GRA administration compared with no treatment both in the corpus (hyperplasia: 0.7 ± 0.2 versus 1.5 ± 0.1; peptic ulcer: 0.9 ± 0.2 versus 1.8 ± 0.3; Table 2) and in the antrum (hyperplasia: 1.5 ± 0.1 versus 2.2 ± 0.3; peptic ulcer: 1.2 ± 0.3 versus 2.1 ± 0.3; Table 3). The pathological changes were also less after GRA administration compared with no treatment (Figure 3(f)). In addition, the inhibitory effects of GRA on the inflammatory responses induced by* H. pylori *infection were almost equivalent to the effect with the antibiotics treatment regime (Figure 3(e)).

**Figure 4:**
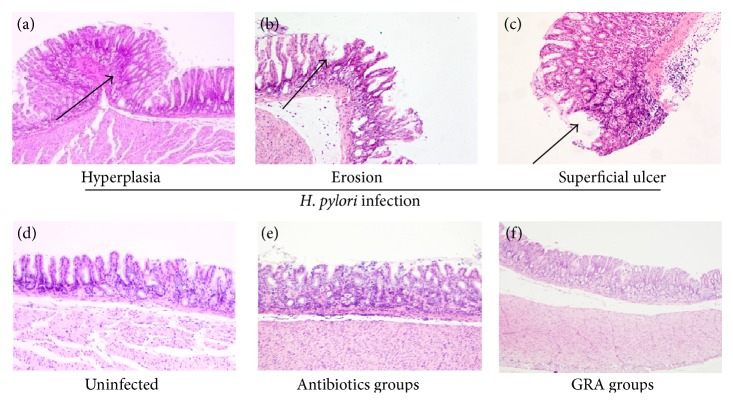
Histopathological analysis of the gastric mucosa in gerbils infected by* Helicobacter pylori *(upper panel: (a) hyperplasia; (b) erosion; (c) superficial ulcer) after antibiotics treatment (e) or 18*β*-Glycyrrhetinic acid (f). Uninfected gerbils were controls (d). H&E; ×40.

Using H&E staining, there was infiltration of mononuclear and polymorphonuclear inflammatory cells into the gastric lamina propria of* H. pylori*-infected Mongolia gerbils (Figure 4(b)). Treatment with GRA significantly decreased the infiltration of neutrophils (corpus: 0.9 ± 0.2 versus 1.6 ± 0.1; antrum: 2.2 ± 0.4 versus 2.9 ± 0.3) and mononuclear cells (corpus: 0.8 ± 0.2 versus 1.3 ± 0.1; antrum: 2.0 ± 0.2 versus 3.0 ± 0.2; Tables 2 and 3) compared with Group B. There were no significant differences in inflammatory cell infiltration rate between treatment with GRA or with antibiotics therapy (Figure 4(c)) compared with the uninfected control Group A (Figure 4(a)).

In* H. pylori*-infected Mongolia gerbils, both the macroscopic mucosal morphology changes and the microscopic pathological changes were more serious in the antrum than the corpus.

### 3.3. GRA Decreased the Expression Levels of COX-2, IL-1*β*, iNOS, and TNF-*α*

**Figure 5:**
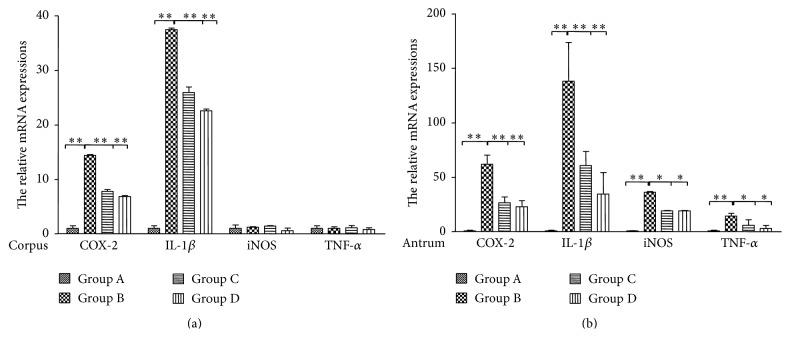
Relative expression levels of COX-2, IL-1*β*, iNOS, and TNF-*α* mRNA in glandular corpus (a) and antrum (b) of Mongolian gerbils infected with* Helicobacter pylori *for 12 weeks and treated with either antibiotics or 18*β*-Glycyrrhetinic acid. Values were set at 1.0 in Group A (uninfected controls) and expressed as mean ± SE relative values. ^*∗*^ *P* < 0.05, ^*∗∗*^ *P* < 0.01.

The expression levels of inflammation-related genes, COX-2, IL-1*β*, iNOS, and TNF-*α*, were detected using qRT-PCR. The results indicated that COX-2 and IL-1*β* were increased 14.4-fold and 37.5-fold in* H. pylori-*infected gastric corpus compared with Group A (Figure 5(a)) and decreased 6.9-fold and 22.6-fold after GRA administration.

Expression of each tested gene was increased in* H. pylori-*infected gastric antrum (Figure 5(b)), and GRA administration significantly decreased the mRNA levels of COX-2 (23.0- versus 61.9-fold), IL-1*β* (34.6- versus 138.2-fold), iNOS (19.2- versus 36.3-fold), and TNF-*α* (3.2- versus 14.4-fold) compared to Group A.

GRA also had similar effects on the cytokine expression compared with the antibiotics, further supported by the H&E analysis.

## 4. Discussion

Gastric cancer is the third leading cause of cancer-related death in the world, and the chronic gastritis induced by* H. pylori* is identified as a high risk factor for this malignancy [5]. In this study, a stable chronic gastritis animal model of* H. pylori *infection in Mongolian gerbils was successfully established with a 100% infection rate. All infected animals developed gastric mucosal hyperemia, edema, and erosion (Figures 3(a)–3(c)), with infiltration of neutrophils and mononuclear cells to the submucosa within 12 weeks of* H. pylori *infection (Tables 2 and 3), which is similar to the disease pattern seen in* H. pylori-*infected patients. The mucosal inflammation and pathological responses were more serious in the gastric antrum than in the corpus, consistent with a previous study [30].

In clinical practice, the common therapy to eradicate* H. pylori* is triple therapy, usually combining a proton-pump inhibitor with two antibiotics, such as clarithromycin, amoxicillin, and omeprazole, which were used in the present study as a positive control (Figure 1). This therapy is not without problems, however, with the combination of clarithromycin and rabeprazole reported to increase the incidence of dissociative disorder [31]; amoxicillin-clavulanic acid therapy can lead to severe side effects and death [32]; omeprazole can induce hypergastrinemia with trophic effects in the stomach [33]. These adverse effects greatly limit the use of these drugs.* H. pylori *have evolved resistance to antibiotics, leading to seriously declining eradication rates. Additionally rates of reinfection and the high costs of antibiotic therapy have led to the idea of using alternative preventive and adjuvant strategies for anti-*H. pylori* therapy produced from natural resources, including plant extracts, plant compounds, and processed plant products.

In a previous study, GRA was found to inhibit the growth of 79.3% of 29* H. pylori* strains [34]. In the present study, although GRA did not eradicate* H. pylori,* it did decrease the intraluminal pH value, which was increased in the untreated* H. pylori *group (Figure 2), and the gastric mucosa hyperplasia, hyperemia, and erosion seen in the untreated* H. pylori *group were much less apparent macroscopically with GRA administration (Figure 3(f)). In addition, the chronic inflammatory response seen in the untreated* H. pylori*-infected group, the infiltration of neutrophils and mononuclear cells, was suppressed in both the corpus and the antrum in the animals treated with GRA (Figure 4). The results indicated that GRA has anti-inflammatory properties and may induce mucosal repairs without eradicating* H. pylori*.

A previous study has shown that increased expression of TNF*α* and IL-1*β* heightens the risk for atrophic gastritis and gastric adenocarcinoma in the early stages of* H. pylori *infection [35]. In the present study, mRNA levels of IL-1*β* and COX-2 were increased in both the* H. pylori*-infected corpus and antrum, while the mRNA levels of TNF-*α* and iNOS were only increased in the* H. pylori*-infected antrum, not in the corpus (Figure 5). The mRNA levels were consistent with the histopathological results that found that infiltration of neutrophil cells and mononuclear cells was more serious in the antrum than in the corpus (Tables 2 and 3) and were also consistent with a previous study in the same species [30]. The occurrence of gastric cancer may be higher in the antrum than the corpus in patients because the inflammation provides a microenvironment that allows cancer cells unlimited growth [36].

Furthermore, the expression of inflammation-related genes, IL-1*β*, COX-2, TNF-*α*, and iNOS, was significantly inhibited by GRA in both the antrum and the corpus (Figure 5), which was consistent with the pathological results. A previous study found that GRA led to the dissociation of a glucocorticoid receptor-HSP90 complex, which blocked inflammation [37]. The results in the present study indicated that the protective effects of GRA were independent of eradication of* H. pylori*. Rather than eradicating the* H. pylori*, GRA diminished* H. pylori*-infected gastritis through the downregulation of inflammation-related pathways. Therefore, long term administration with GRA was useful in preventing gastric lesions, repairing the gastric mucosa, modulating the microenvironment that affects gastric cell growth and proliferation, and further decreasing the possibility of gastric tumorigenesis.

GRA has also been reported to inhibit superoxide anion formation and the level of reactive oxygen species as an antioxidation compound [38]. A further published paper showed that GRA elicited NAG-1, a target for nonsteroidal anti-inflammatory drugs, which is overexpressed in various cancer cells [39]. This work suggests that the combination of GRA and nonsteroidal anti-inflammatory drugs may confer a benefit in the treatment of gastritis and gastric cancer, and GRA might enhance the anticancer drug effect as an adjuvant.

## 5. Conclusion

GRA showed marked inhibitory effects in* H. pylori*-infected gastritis in gerbils, leading to the proposal that GRA may be a pivotal bioactive inhibitor of gastric inflammation; it might be a potential nutraceutical for remedying inflammation, a chemoprotector for gastric cancer, and a valuable adjuvant for antibiotic treatments.

## Figures and Tables

**Table 1:** PCR primers used for real-time quantitative RT-PCR analysis of the genes active in *Helicobacter pylori* infection of the gastric mucosa.

Genes		Primer sequence	*T*_*m*_	Product size (bp)
GADPH	F	5′-AACGGCACAGTCAAGGCTGAGAACG-3′	81.00	118
	R	5′-CAACATACTCGGCACCGGCATCG-3′		

IL-1*β*	F	5′-TGACTTCACCTTGGAATCCGTCTCT-3′	78.50	91
	R	5′-GGCAACAAGGGAGCTCCATCAC-3′		

TNF-*α*	F	5′-GCTGCCCCCACCTCGTGCTC-3′	82.50	89
	R	5′-CTTGATGGCAGACAGGAGGCTGACC-3′		

COX-2	F	5′-GCCGTCGAGTTGAAAGCCCTCTACA-3′	80.50	97
	R	5′-CCCCGAAGATGGCGTCTGGAC-3′		

iNOS	F	5′-GCATGACCTTGGTGTTTGGGTGCC-3′	81.00	110
	R	5′-GCAGCCTGTGTGAACCTGGTGAAGC-3′		

*T*_*m*_: annealing temperature.

**Table 2:** Histopathological response seen in gerbil gastric corpus in response to *Helicobacter pylori* infection and treatment with either antibiotics therapy or 18*β*-Glycyrrhetinic acid (GRA).

Groups	Treatments	Corpus			
		Infiltration of neutrophils	Infiltration of mononuclear cells	Hyperplasia	Peptic ulcer
A	Uninfected	0.0 ± 0.0	0.0 ± 0.0	0.0 ± 0.0	0.0 ± 0.0
B	*H. pylori *infection	1.6 ± 0.1	1.3 ± 0.1	1.5 ± 0.1	1.8 ± 0.3
C	Antibiotics	0.9 ± 0.2	0.7 ± 0.2^*∗*^	0.6 ± 0.3^*∗*^	0.8 ± 0.2^*∗*^
D	GRA	0.9 ± 0.2^*∗*^	0.8 ± 0.2^*∗*^	0.7 ± 0.2^*∗*^	0.9 ± 0.2^*∗*^

^*∗*^ *P* < 0.05, compared with Group B. Values for results are expressed as means ± SD.

**Table 3:** Histopathological response seen in gerbil gastric antrum in response to *Helicobacter pylori* infection and treatment with either antibiotics therapy or 18*β*-Glycyrrhetinic acid (GRA).

Groups	Treatments	Antrum			
		Infiltration of neutrophils	Infiltration of mononuclear cells	Hyperplasia	Peptic ulcer
A	Uninfected	0.0 ± 0.0	0.0 ± 0.0	0.0 ± 0.0	0.0 ± 0.0
B	*H. pylori* infection	2.9 ± 0.3	3.0 ± 0.2	2.2 ± 0.3	2.1 ± 0.3
C	Antibiotics	2.0 ± 0.4^*∗*^	1.6 ± 0.1^*∗*^	1.6 ± 0.3^*∗*^	1.0 ± 0.0^*∗*^
D	GRA	2.2 ± 0.4^*∗*^	2.0 ± 0.2^*∗*^	1.5 ± 0.1^*∗*^	1.2 ± 0.3^*∗*^

^*∗*^ *P* < 0.05, compared with Group B. Values for results are expressed as means ± SD.
